# Examining the Effect of Host Recruitment Rates on the Transmission of *Streptococcus suis* in Nursery Swine Populations

**DOI:** 10.3390/pathogens9030174

**Published:** 2020-03-01

**Authors:** Elissa Giang, Benjamin M. Hetman, Jan M. Sargeant, Zvonimir Poljak, Amy L. Greer

**Affiliations:** 1 Department of Population Medicine, University of Guelph, 50 Stone Rd E, Guelph, ON N1G 2W1, Canada; bhetman@uoguelph.ca (B.M.H.); sargeanj@uoguelph.ca (J.M.S.); zpoljak@uoguelph.ca (Z.P.); 2 Center for Public Health and Zoonoses, University of Guelph, 50 Stone Rd E, Guelph, ON N1G 2W1, Canada

**Keywords:** *Streptococcus suis*, swine, batch management systems, mathematical modeling, epidemiology, recruitment rate, transmission dynamics

## Abstract

*Streptococcus suis* is a swine pathogen that is capable of causing severe outbreaks of disease in the nursery. Demographic parameters such as host recruitment rates can have profound effects on the transmission dynamics of infectious diseases and, thus, are critically important in high-turnover populations such as farmed swine. However, knowledge concerning the implications that such parameters have on *S. suis* disease control remains unknown. A stochastic mathematical model incorporating sub-clinically infected pigs was developed to capture the effects of changes in host recruitment rate on disease incidence. Compared to our base model scenario, our results show that monthly introduction of pigs into the nursery (instead of weekly introduction) reduced cumulative cases of *S. suis* by up to 59%, while increasing disease-removal rates alone averted up to 64% of cases. Sensitivity analysis demonstrated that the course of infection in sub-clinically infected pigs was highly influential and generated significant variability in the model outcomes. Our model findings suggest that modifications to host recruitment rates could be leveraged as a tool for *S. suis* disease control, however improving our understanding of additional factors that influence the risk of transmission would improve the precision of the model estimates.

## 1. Introduction

*Streptococcus suis* is a production disease of swine and one of the most common causes of piglet morbidity and mortality after weaning [1]. Disease resulting from infection with *S. suis* usually occurs in piglets up to 10 weeks in age, although pigs of any age can be affected [1]. Cases may present as severe systemic infections such as meningitis, septicemia, arthritis, pneumonia, and sudden death [2]. The bacterium commonly resides in the upper respiratory tract of pigs and is highly diverse, with 35 serotypes known to date [3]. Among the 35 serotypes, serotype 2 is often associated with disease in pigs and is most frequently isolated from diseased cases [3]; however, not all strains are pathogenic, while varied virulence can exist among pathogenic strains [4]. Pigs harboring *S. suis* may present as various manifestations including sub-clinical infection, clinical-infection, or a carrier state upon recovery of infection [1]. Piglets can become exposed to *S. suis* from vaginal secretions during or after parturition, while carriers (both sub-clinical and clinical) represent a possible source of infection for their pen mates after they are mixed with other piglets in the nursery [1,5]. 

Horizontal transmission of *S. suis* primarily occurs through the oro-nasal route and colonizes the tonsils of both clinically ill and healthy pigs [6,7]. Moreover, increasing evidence of aerosol exposure suggests that airborne transmission is an important route for *S. suis* spread across short distances [8,9,10,11,12]. Outbreaks of *S. suis* in the nursery are frequently credited to the introduction of a sub-clinically infected pig into the herd [13,14]. However, the association between carrier rates and disease occurrence in the nursery remains unknown despite their importance in *S. suis* epidemiology [5,14,15]. Strategies for effective disease control for *S. suis* remains challenging due to the low reported success rates of conventional disease control programmes. For instance, antibiotic prophylaxis has been shown to be effective in eradicating other endemic swine pathogens such as *Actinobacillus pleuropneumoniae*, *Mycoplasma hyopneumoniae*, and *Lawsonia intracellularis* in swine herds [16,17,18]. However, eradication efforts have been unsuccessful in eliminating the carrier state of *S. suis*, since pigs are colonized very early after birth [19,20]. *S. suis* vaccine use on farms remains uncommon. A 2006 report from the National Animal Health Monitoring System reports that <7% of herds in the U.S. use a *S. suis* vaccine [21]. Vaccination efficacy is often hampered by the inability to eliminate local tissue invasion and carrier states in pigs as well as the inability to offer cross-serovar protection [22,23,24,25]. Thus, alternative approaches for effective disease control in the nursery would be beneficial. 

Strategies for optimal disease control require a comprehensive understanding of the mechanisms by which pathogens can invade and propagate. The extent to which a pathogen can be maintained within a population depends on two key components: (i) the transmissibility of the pathogen and (ii) the availability of susceptible hosts [26]. The transmission potential of a pathogen (also known as the basic reproductive number, *R_0_*) consists of three key factors that influence the spread of disease: the rate of contact between susceptible and infectious individuals (*c*), the probability of transmission given a suitable contact (*p*), and the duration of infectiousness (*D*) [27]. Attempts to influence these factors to disrupt pathogen transmission have been previously investigated for *S. suis*. For example, the application of early segregated weaning techniques failed to eliminate the carrier state of *S. suis* since the bacterium is an early colonizer of pigs [19]. Similarly, Dekker et al. [9] demonstrated that spatial separation of pigs (at the pen-level) would not be sufficient in preventing the spread of the *S. suis* in either directly or indirectly exposed pigs due to the rapid colonization of the bacterium. However, the authors suggested that spatial separation at the herd-level may reduce the risk of transmission [9].

In the absence of effective methods to control this disease, infectious disease theory suggests that efforts could be focused on limiting the availability of susceptible hosts by modifying host population demographics. The influence of host recruitment rates (number and time of entry of susceptible hosts into the population) in disease spread has been well-established in human infectious disease epidemiology. For example, a comparative analysis of the persistence of measles in cities and on islands found that large and growing populations in dense cities supported the continued propagation of the virus, whereas “breaks” in the continuity of measles transmission were found in smaller island communities, which helped limit the transmission of the disease [28]. This study and others [29,30] have suggested that increased breaks in a system without continuous supply of susceptible individuals may reduce infectious numbers or increase the chance of stochastic fade-out and subsequent elimination of the infection. 

In the context of swine production systems, management practices often dictate both the supply of susceptible hosts into the system and the contact patterns between them. For example, the timing of new births in the population are often tightly controlled events that determine the influx of susceptible pigs downstream in production; while herd management will affect the relative contact of animals between different groups (i.e., continuous flow, all-in/all-out systems). For swine diseases such as *S. suis*, understanding the underlying mechanisms for disease transmission can be critically important for implementing and optimizing disease control strategies. Changes in host recruitment rates can be modified with the use of batch management systems (BMS), which is a practice that allows for modifications in the farrowing interval (fixed breeding and farrowing) and the delivery of batches of pigs segregated by time [31]. These systems allow for “breaks” in the timing of the introduction of new susceptible pigs into the nursery and the ability to clean and disinfect rooms between batches of pigs. BMS have been previously shown to be effective in reducing the incidence of other swine diseases [32], however the implications that BMS may have on *S. suis* disease control have not been previously examined.

Mathematical models are useful tools for understanding disease dynamics because they allow us to test explicit assumptions about the hypothesized mechanisms leading to the spread of pathogens and to explore scenarios *in silico* (via computer simulation) [26]. The goal of the study was to use mathematical modeling to simulate disease control strategies that swine producers may practically employ to reduce the spread of *S. suis* in the nursery. Based on a nursery barn that experienced a 6-month outbreak of *S. suis* as a case study, we developed a stochastic mathematical model to describe *S. suis* transmission within the nursery for a farrow-to-finish swine farm in Ontario, Canada. The objective of this study was to examine whether modifications to the number and timing of susceptible hosts (i.e., newly weaned piglets) entering into the nursery could be used as a management tool for controlling *S. suis* disease outbreaks in the nursery.

## 2. Results

### 2.1. Model Fit 

**Figure 1:**
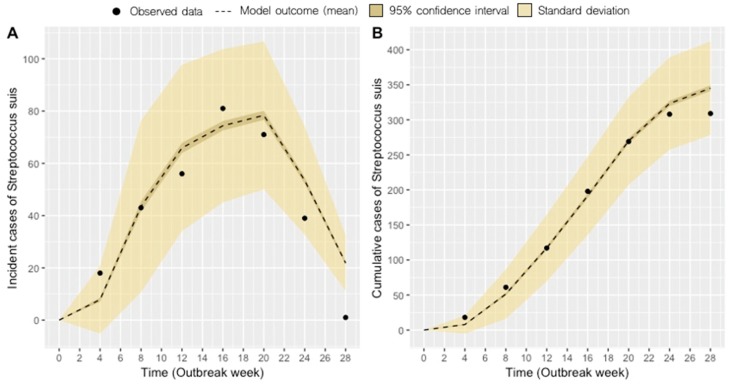
Average model fit (dashed line) with a 95% confidence interval (gold bands) to monthly incident case data **(A)** and average model fit to monthly cumulative case data with a 95% confidence interval (gold bands) **(B)** after 1000 model simulations.

The goal of maximum likelihood estimation is to find model parameter values that describe the distribution that maximizes the probability of observing the empirical data. After fitting our model to the observed outbreak data, our best-fit estimates for *D*, *σ*, and *p* were 1.43 weeks, 4.8 weeks, and 0.22, respectively. The model graphically appears to have good agreement with the observed monthly incidence and cumulative case data (Figure 1).

### 2.2. Intervention Scenarios 

**Figure 2:**
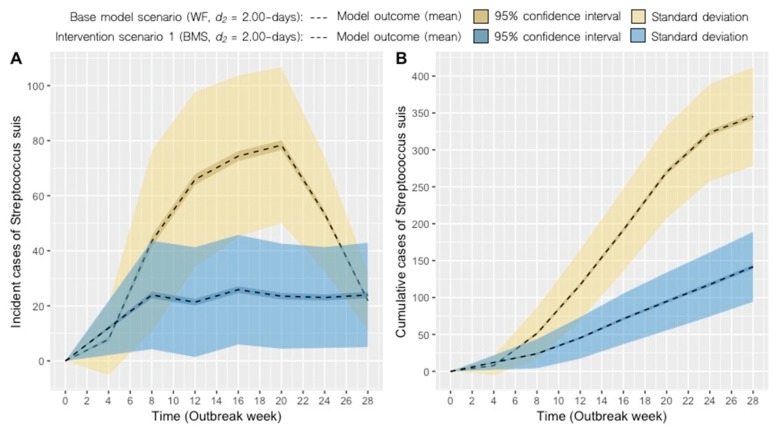
Scenario 1: Predicted incident cases (**A**) and cumulative cases (**B**) of *S. suis* in the nursery using a batch management system (BMS) with a 2.00 day disease-removal rate (*d_2_*) of clinically infected pigs (blue) compared to the base model scenario (yellow).

**Figure 3:**
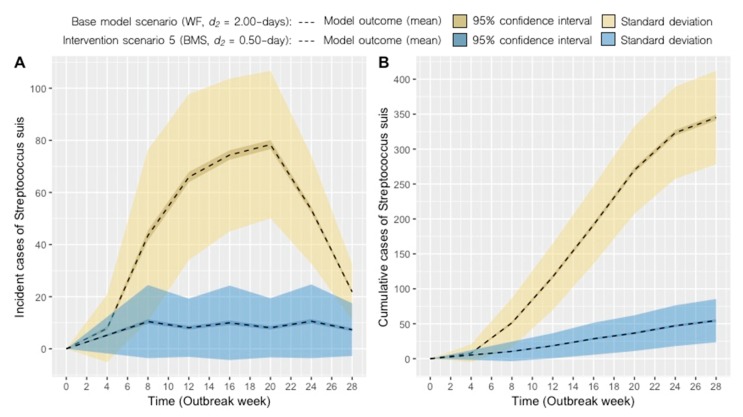
Scenario 5: Predicted incident cases (**A**) and cumulative cases (**B**) of *S. suis* in the nursery using a monthly batch management system (BMS) with a 0.50 day disease-removal rate (*d_2_*) of clinically infected pigs (blue) compared to the base model scenario (blue).

**Figure 4:**
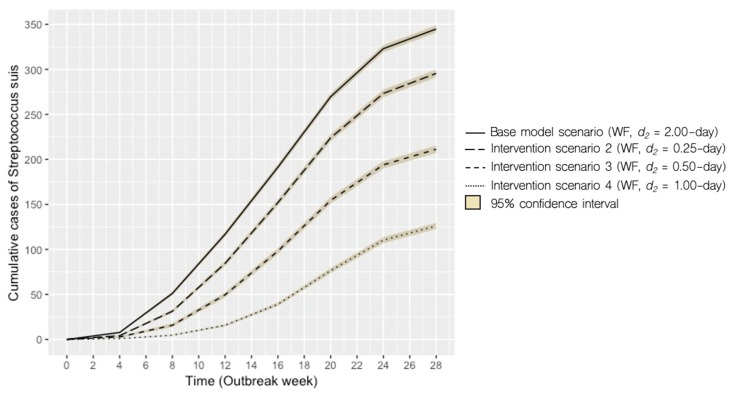
Scenario 2 to 4: Predicted cumulative cases of *S. suis* in the nursery using a weekly-farrowing (WF) system with disease-removal rate (*d_2_*) of 2.00 days as the base model scenario compared to scenarios 2 to 4 with varying disease-removal rates (*d_2_*).

Model simulations of the impact of different management strategies (interventions) are presented. The results are presented graphically with each intervention compared against the base model scenario based on 1000 model simulations per scenario. Figure 2 depicts the transition to a BMS with the disease removal parameter held constant (i.e., *d_2_* = 2.00 days) and cumulative cases reduced from the base model scenario. Similarly, Figure 3 presents the combined effect of a BMS and more rapid removal of clinically ill pigs (i.e., *d_2_* = 0.50-day). The potential impact of increased clinical monitoring and removal of clinically ill pigs is shown in Figure 4, with scenarios 2 to 4 under a Weekly farrowing (WF) system. Model outputs of predicted incident and cumulative cases of *S. suis* for each scenario are compared in Table 1. 

### 2.3. Projected Maximum Cumulative Case Numbers 

**Figure 5:**
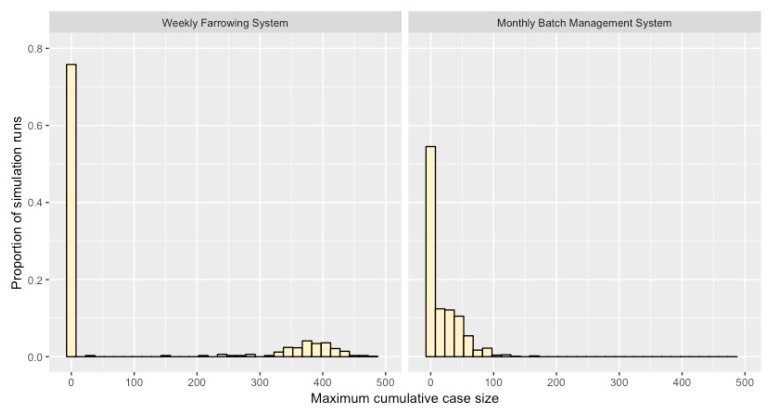
Histogram of proportion of simulation runs (n = 1000) resulting in a range of *S. suis* cumulative cases for the weekly farrowing system and monthly batch management system.

Under the base model scenario, the results of 1000 simulation replicates showed that 24% of the model simulations resulted in subsequent transmission of the disease (clinically-infected cases > 1). We found that the largest observed outbreak out of 1000 simulations was an outbreak with 476 clinically-infected cases, which is equivalent to 23.8% (476/2000) of the herd infected under the base model scenario (Figure 5). The transition to a BMS (scenario 1) showed an increased probability of subsequent transmission (relative to the base model) in the population with 58% of model simulations that resulted in clinically-infected cases. Moreover, the largest reported outbreak size based on 1000 simulation replicates was 158 clinically-infected cases, or 7.9% (158/2000) of the herd infected (Figure 5).

### 2.4. Sensitivity Analysis 

**Figure 6:**
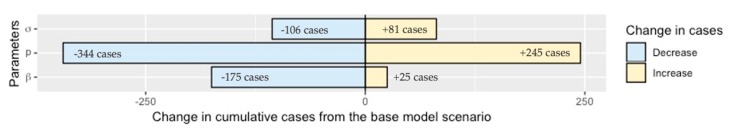
Tornado plot of the results from the sensitivity analysis of the latent period (*σ*), probability of sub-clinical pigs becoming clinically infected (*p*), and the transmission coefficient (*β*) on cumulative cases as the model output. Changes in the cumulative cases of *S. suis* are compared against the base model scenario (vertical black line), with increases in cumulative cases depicted in yellow and decreases depicted in blue, with the case range reported.

**Figure 7:**
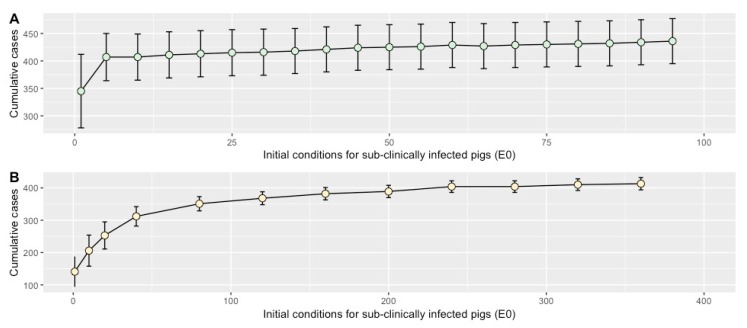
Sensitivity analysis of initial conditions for sub-clinically infected pigs (*E0*) in a weekly farrowing system (**A**) and batch management system (**B**) with changes observed in cumulative cases and 95% confidence interval based on 1000 model replicates.

Model outputs were the most sensitive to the probability of a sub-clinically infected pig developing clinical signs (*p*), while the transmission coefficient (*β*) and latent period (*σ*) were moderately sensitive, as shown in Figure 6. Changes in the initial conditions for *E0* in the WF model did not appear to dramatically change the maximum outbreak size, with cases increasing accordingly with higher *E0* values (Figure 7A). Under a BMS, model projections estimated that low initial values of *E0* (under 80 pigs) appeared to be effective in reducing cumulative case counts compared to the WF base model scenario (i.e. *S0* = 100, *E0* = 1, *I0* = 0). However, initial *E0* values ≥ 80 in a BMS resulted in similar behavior to that observed in the WF system, with higher values of *E0* associated with a larger final outbreak size (Figure 7B). 

## 3. Discussion

Our results have important implications for the control of *S. suis* and highlight important areas of uncertainty where more research is required. Given that there is no consistently effective vaccine against *S. suis* and treatment options are increasingly limited [33], identifying biosecurity practices that may limit the spread of this economically important disease is of great value.

Our model findings showed that changes in the host recruitment rate, particularly in populations with high turnover of animals, can have substantial effects on *S. suis* disease dynamics and the resulting transmission between pigs. Designed to mimic the management practices on this specific case-study farm, the base model scenario showed overall good agreement with the observed case data. This suggests that the rapid replenishment of susceptible pigs into the nursery may be an important factor in driving outbreaks of disease. Under a BMS scenario, the increased timing between entry of pigs into the nursery appeared to consistently decrease the number of cumulative cases of *S. suis*. This may be explained by the exhaustion of susceptible hosts in a population to a level where disease transmission is reduced or can no longer be maintained. These findings are consistent with a number of other mathematical modelling studies in various host-pathogen systems that have previously examined the relationship between host recruitment rates (i.e., influx of new susceptible hosts into the population) and peaks in disease outbreaks [34,35].

The monthly BMS scenario showed that segregation between batches of pigs was an effective practice for reducing the cumulative number of *S. suis* cases in this nursery. However, it is important to note that transition to a BMS did not appear to reduce the number of *S. suis* incident cases in the nursery, which might suggest that low levels of disease may persist under a BMS relative to a WF system (Figure 2A and Figure 3B). While we did not have previous incidence estimates of observed *S. suis* cases prior to this documented outbreak for this farm, we interpret these as baseline levels of expected *S. suis* cases that did not result in an outbreak. Given the endemic nature of this bacterium on swine farms worldwide [36], it would be unrealistic to assume that eradication of *S. suis* is currently possible. Moreover, since transmission can still occur between pigs within the same batch, it is unsurprising that cases would still arise in a BMS. Further analysis of model simulations showed that while the BMS scenario (scenario 1) yielded a higher probability of an outbreak occurring, cumulative counts of *S. suis* were often smaller relative to the WF base model scenario (Figure 5). Thus, our results demonstrate that while transition to a BMS cannot prevent within-herd transmission of *S. suis*, a significant reduction in clinical cases observed may be possible with batch-level separation. 

Our model also highlighted the importance of an additional management-influenced parameter (i.e., *d_2,_* disease-removal rate of clinically infected pigs). Under coupled interventions (scenario 5), the model projected an 84% reduction in cumulative cases compared to the base model scenario (Figure 3B). When different disease-removal rates were assessed in a WF system, the model showed that this practice alone could contribute to a substantial decrease in cumulative cases of *S. suis* (Figure 4). Recognizing that transition to a BMS can be complex, time-consuming, or simply incompatible with the design of the production facility/farm, our model suggests that early removal rates of clinically infected pigs may be a viable option for reducing further cases of disease in the event that a BMS cannot be implemented. Here, we defined early removal to include the development of protocols that help with identification of sick pigs in acute or chronic stages of *S. suis* infection, followed by appropriate removal or segregation of diseased pigs placed in a hospital pen, where no contact with the rest of the herd can be achieved. 

While the BMS appeared to be effective in reducing the overall prevalence of *S. suis* cases, it is important to highlight that the results reported here are dependent on several model assumptions that are important to consider for future investigation. We simplified the model to consider the clinical course of *S. suis* infection; yet we understand that sub-clinical pigs likely contribute a sizable fraction of infection [1]. Previous studies conducted have been unable to correlate the rates of sub-clinical carriage in a herd and the observed diseased cases [13,14]. As a result, there is limited knowledge of their relative contribution to *S. suis* transmission. Moreover, identification of sub-clinically infected pigs is challenging since these pigs do not display obvious signs of infection, which therefore requires making some simplifying assumptions given the knowledge gap. As a result, our model may under- or over-estimate the model outcomes depending on the role that sub-clinical pigs play in transmission. 

Variability in the model was assessed using a sensitivity analysis to examine changes in our model outputs under different ranges of key parameter values and also using different assumptions regarding the initial model conditions. The highest variability in model outputs was observed in the probabilistic parameter *p* (the probability of developing clinical signs) (Figure 6). While we can expect that higher probabilities are associated with more cases of clinical disease, the true value of *p* is likely influenced by several factors related to the host, pathogen, and environment. For instance, external factors that induce stress in piglets (e.g., weaning, mixing with other litters, overcrowding, and poor housing conditions) have been shown to correlate with increased clinical infections in the nursery [1,37,38]. Additionally, host-specific factors related to genetics, age, and immune status can also play a role in disease onset [39]. At the pathogen-level, variation in *p* may also be attributed to the varied virulence in pathogenic strains of *S. suis*. Serotype 2 is the most commonly associated with disease in pigs, however not all strains of serotype 2 are pathogenic, and virulence can vary among strains within the serotype [4]. However, the extent of these relationships is complex and warrants increased attention to the continued collection of empirical data to improve the model assumptions in this area.

Results from the sensitivity analysis of the initial model conditions showed that under a WF system, the ranges assessed for values of *E0* did not dramatically change the model outputs with the final outbreak size increasing when *E0* was increased (Figure 7A). However, under a BMS, low values of *E0* resulted in lower cumulative cases compared to the WF system. Interestingly, when values of *E0* were increased in the BMS scenario, the system behaved similar to the WF observations (Figure 7B). If the true value of *E0* is high in most settings, a BMS intervention would likely not be any more effective in reducing cases compared to the WF system; however, if *E0* is low, then a BMS may be a practice worth considering to reduce disease burden in the nursery. While the true value of *E0* is not known for this system, this additional analysis emphasizes the need for additional data in this area to provide more precise estimates and recommendations. 

Inherent to these types of models, our study has limitations that are important to consider for future work. First, while *S. suis* may be introduced into the herd through multiple routes, our model only considered the introduction of *S. suis* into the herd via sub-clinical infected pigs. Previous studies have detected *S. suis* serotype 2 in both specific pathogen free (SPF) and hysterectomy-derived herds [40,41]. The authors suggest that these infections were likely introduced through contamination of the environment, personnel, or equipment. Mechanical vectors including mice and houseflies have also been implicated as vehicles of *S. suis* transmission; while feces, contaminated feed, water, work boots, and needles have also been shown to transmit the bacterium [1,42]. Breeding rooms may also serve as a potential source of transmission, similar to *Salmonella* infections in pigs [43]. While such introductions can occur, *S. suis* is endemic on most swine farms worldwide and the detection of *S. suis* on farms is likely not a good predictor for strains of clinical relevance. As such, our model focused on the introduction of a sub-clinical infected pig, which has been reported to play an important role in outbreaks of *S. suis* [13,14,40]. 

As described above, sub-clinically infected pigs are an important component in *S. suis* epidemiology and may serve as a potential reservoir of infection for susceptible pigs [13]. However, the relative contribution of these pigs to the incidence of *S. suis* cases remains unknown since the carrier state is not a good indicator of clinical disease in a herd. A study conducted by Clifton-Hadley et al. [40] reported herds with nearly 100% carrier rates of *S. suis*, but reported that less than 5% of the herds were observed to have apparent disease. In the absence of sufficient data, we simplified the model to only consider the clinical course of *S. suis* infection (i.e., only clinically infected pigs are infectious). However, given their importance, we attempted to represent the relationship as a probability in the model, such that each new sub-clinical infected pig has the probability of developing clinical signs and is infectious (*p*) or remains sub-clinically infected (1 – *p*); this parameter (*p*) was determined by model fitting, which was deemed appropriate since *S. suis* carriage is reported to vary across herds [1,5].

We did not include batch-to-batch carryover of *S. suis* in the BMS model. While the working environment on farms may serve as a potential infection source for pigs [1,42], we were limited by available data to incorporate this potential route of transmission. In reality, cleaning and disinfection are performed between batches of pigs, with *S. suis* generally being susceptible to most disinfectants and readily inactivated using hot water and exogeneous heat sources >55 °C [42]. To simplify our model, we assumed that disinfection and cleaning performed between batches of pigs is effective in reducing the microbial load in the environment to sufficiently low levels, such that transmission via this route was considered negligible. While bacterial carry-over from a previous batch may be possible in sustaining *S. suis* transmission, further research is required to determine the number of viable bacteria in the environment, which could be used to inform initial conditions and parameter values using an environmental compartment.

We have ignored the potential impact of herd structure on transmission in both WF and BMS models. This is principally a consequence of the limited availability of data on how pigs were managed within this study during the time of the outbreak. Under a WF system, we assumed homogeneous mixing of pigs (i.e., pigs in the same room or in different rooms have the same probability of contact with a clinically infected pig), which does not account for heterogeneity in host contacts. Transmission experiments have shown that spread of *S. suis* serotype 9 within- and between-pens does occur, with transmission rates lower for between-pen transmission [9]. In addition to transmission through direct and indirect contact between pens, there would be other means of transmission between groups of pigs some distance apart, for example through fomites [42] or airborne spread [8,12]. However, estimating the transmission rate for each route using mortality data is not possible. Similarly, the decision to treat the BMS model as a fully closed system (no mixing between batches) was necessary in order to maintain model parsimony and avoid making additional assumptions in the absence of sufficient data. Consequently, it is difficult to assess what impact neglecting a more complex herd structure would have on our model outputs. Previous studies have examined disease dynamics in smaller structured populations using a metapopulation approach [44]. Alternatively, models could also be stratified by age or level of risk to capture heterogeneity in host contacts. These types of model structures may be necessary for future modelling studies to fully understand the impact of these mixing assumptions.

## 4. Materials and Methods 

### 4.1. Case Study Data

**Figure 8:**
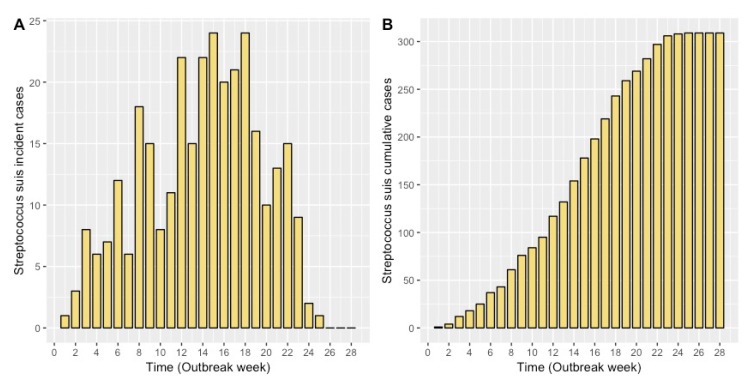
(A) Epidemic curve of *Streptococcus suis* incident cases and (B) cumulative cases (*n* = 309) within a nursery of a farrow-to-finish swine farm from 6 October 2011 and 30 March 2012 in Ontario, Canada.

Retrospective mortality data from a 250-sow farrow-to-finish swine farm (Ontario, Canada) that experienced a *S. suis* outbreak in the nursery over a 6-month time period were used for this study. Descriptive details of this dataset have been previously described [45]. Briefly, the outbreak occurred from October 6, 2011 to March 30, 2012 and involved 20 cohorts of weaned pigs. Mortality data during this period included all-cause mortality; however, we assumed that the majority of cases were due to *S. suis* based on clinical signs of acute meningitis. Laboratory confirmation and post-mortem examination confirmed 12 clinical cases of meningitis due to *S. suis* serotype 2. At the time of the original outbreak, the herd was managed according to a weekly farrowing (WF) system, with a total of 20 sow groups, each group farrowing on a weekly basis. On average, 100 new susceptible pigs entered the nursery each week (up to 20 weeks) and were housed all-in/all-out (AIAO) by room. From the dataset, we extracted case-based records over a 28-week period (duration of the outbreak) and generated a weekly-time series of case counts (incident cases) and cumulative cases for our study analyses (Figure 8). 

### 4.2. Model Structure

All model development and analyses were performed in the statistical programming language R version 3.5.2 [46]. A stochastic compartmental model was constructed to represent the management of the study farm population using a susceptible, exposed, infectious (*SEI*) framework. We opted to use a stochastic model, rather than a deterministic model to account for biological variability in small population sizes and in the clinical course of *S. suis* infections. The model simulates the infection process as a series of random events with respect to time. Model events were implemented stochastically, with each event independent of the previous event. Pigs in the model could be classified as being in one of three possible infection states (which were mutually exclusive):

- *S*: represents animals that are able to acquire infection from an infectious pig (via direct contact and/or airborne transmission).
- *E*: represents animals that are sub-clinically infected (non-infectious carriers).
- *I*: represents animals that are clinically-infected with disease signs (infectious to others).

### 4.3. Model Transitions and Initial Conditions 

**Figure 9:**
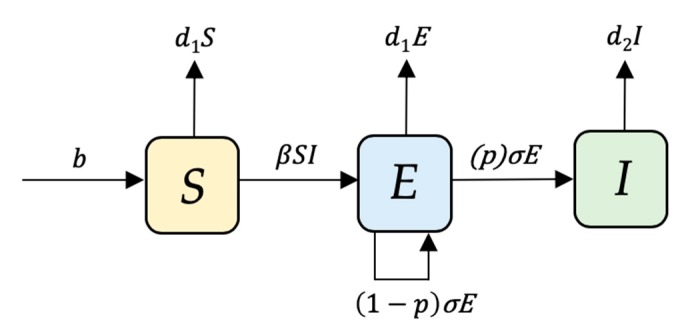
Susceptible-exposed-infectious (*SEI*) compartment diagram describing *Streptococcus suis* transmission within a nursery pig population driven by the influx of susceptible pigs (*b*) and the presence of sub-clinically infected pigs (*E*) with the probability of becoming clinically-infected (*p*) and transitioning to an infectious state (*I*) or remaining sub-clinically infected with probability (1–*p*).

The movement of susceptible pigs (*S*) to the Exposed (*E*) class is governed by the transmission coefficient (*β*). After a latent period (*σ*^−1^), exposed pigs can transition to a state of infectiousness (*I*), with probability (*p*), or remain sub-clinically infected with a probability of (1 – *p*). Population demographics are included in the model, where susceptible pigs initially enter the population at a constant recruitment rate (*b*). The recruitment rate was modelled as a constant, based on the operational logistics of a WF system on this farm during the time of the outbreak. Susceptible and sub-clinical infected pigs were removed from the population at a constant rate based on the average time spent in the nursery on this farm, (*d_1_*); while clinically infected pigs were removed from the population by the disease-removal rate (*d_2_*). The dynamics of the system described are presented in Figure 9 and are captured by a series of differential equations:

*dS*/*dt* = *b* – *βSI – d_1_S,* (1)

*dE/dt =βSI + (1 – p)σE – p(σE) – d_1_E,* (2)

*dI/dt =p(σE) – d_2_I.* (3)

Transitions between different infection states occurred in a probabilistic manner according to a Poisson process. The corresponding events and transitions are shown in Table 2, while parameter inputs are displayed in Table 3. 

To start the initial infection process, we populated the nursery with susceptible pigs (*S0* = 100) and assumed that *S. suis* is introduced into the population by a sub-clinically infected pig (*E0* = 1). This was deemed reasonable, since these pigs do not display clinical signs and are not likely to be identified as “infected” and thus, remain in the nursery [5]. In contrast, clinically infected pigs who display disease signs are less likely to go unnoticed prior to entry into the nursery, therefore (*I0* = 0). To model the WF system, we allowed for 100 new susceptible piglets to enter the nursery each week (up to 20 weeks), which was seeded with a single sub-clinically infected pig each week. In the WF system, we assumed that there is homogeneous mixing of pigs within and between rooms (i.e., each pig has the same probability of being infected by a clinically infected pig regardless of room separation). Transmission via infectious aerosols across short distances has been documented in both field and experimental studies of *S. suis* in pigs [8,9,10,11,12] and subsequently justified our mixing assumptions. To capture this, we allowed for contact between infectious pigs of one group and susceptible pigs in the next group that entered the nursery (open system).

### 4.4. Model Assumptions

The model assumes that all pigs entering the nursery are susceptible to infection with a pathogenic strain of *S. suis*. The model also assumed that *S. suis* is introduced into the herd by a single sub-clinically infected pig. While other transmission routes are possible (e.g., contaminated equipment, flow of works, pest/rodent etc.), they were not considered in our model due to limited data availability. We assumed that only clinically infected pigs can infect susceptible pigs. Given the limited understanding of the effects of sub-clinically infected pigs on *S. suis* disease occurrence, we only considered the clinical course of infection in the absence of sufficient data. Further, *S. suis* infections are known to be multi-factorial, where factors related to the host, pathogen, and system can impact the occurrence and severity of disease or morbidity in pigs [37]. As a result, there are limited empirical data on parameters that determine the natural course of this disease, therefore these parameters were estimated using model fitting. 

### 4.5. Model Fitting

The model was fit to the monthly cumulative cases using an iterative procedure to estimate the unknown parameters (*D*, *σ,* and *p*). This was done using maximum likelihood estimation using the log-likelihood function in R [46], where we examined a range of plausible parameter values to determine best fit values that maximized the likelihood of the model and the data. Using our previously estimated *R_0_* value (1.4) for this outbreak [47], we determined the transmission coefficient (*β*) based on the mathematically known relationship for micro-parasitic infections [27] and our best-fit value for the infectious period (*D*):

*β* = *R_0_/D*. (4)

We assessed the fit of the model output to the observed monthly incident and cumulative case counts using maximum likelihood and confirmed this by visual inspection. 

### 4.6. Base Model and Intervention Scenarios 

The model using our best-fit parameters served as the base model scenario (described in Section 4.2 to Section 4.3) for comparison to our model interventions. To examine the influence of modifications in management practices in the nursery, we explored five intervention scenarios using the maximum cumulative case number as the outcome measure against the base model scenario. A description of all scenarios can be found in Table 4. In scenario 1, we simulated the effect of a monthly BMS, such that 400 new susceptible piglets entered the nursery every 4 weeks (up to 20 weeks) and were seeded with a single sub-clinically infected pig. Assuming that the facility is required to produce an equivalent number of pigs and has the facility space to accommodate a larger group of animals, we increased the batch size proportional to the average number of pigs that entered on a weekly basis. In the BMS scenarios, we assumed homogeneous mixing of pigs within the same batch but did not allow for mixing of pigs in different batches. In practice, a BMS would typically allow for cleaning and disinfection between batches of pigs [31], therefore we ignored the potential for between-batch carryover, where each batch is treated as a closed system. In scenarios 2 to 4, we evaluated the influence of changes in the early disease removal rate (*d_2_*) by examining a range of reasonable removal times for clinically infected pigs (0.25 day, 0.50 day, 1.00 day), assuming that the facility can increase monitoring for clinical signs in the nursery. Lastly, scenario 5 examined the influence of coupled interventions (i.e., BMS and early disease removal rates, *d_2_* = 0.50 day). 

For both our base model and intervention scenarios, the events were implemented stochastically using the “ssa.exact.function” as part of the *adaptivetau* package in R [46,48]. This function implements Gillespie’s direct method [49] and assumes that events are independent. For each simulation, a total of 1000 model simulations were performed using a random seed and the model simulation ran for 28 weeks (the duration of the original outbreak). Model simulations that did not result in an outbreak (no subsequent disease transmission) were filtered out to provide comparable model projections against our empirical outbreak data. For simulation runs that resulted in outbreaks (e.g., at least 1 case), the model simulation outcomes (weekly and cumulative incidence) were averaged and we derived the 95% confidence interval around the average number of incident and cumulative cases and standard deviation to account for stochastic variation in our model outcomes. The model outputs were aggregated by month to allow for comparison of our base model and intervention scenarios.

### 4.7. Simulations of Maximum Cumulative Case Numbers 

For the base model scenario and BMS scenario (scenario 2), 1000 model simulations were examined. For each scenario, we derived the proportion of all model simulations that resulted in i) no subsequent transmission (i.e., no outbreak), and ii) subsequent transmission resulting in an outbreak (i.e., secondary transmission after introduction). Histograms were plotted to examine the overall probability of an outbreak occurring and the distribution of cumulative case sizes expected.

### 4.8. Sensitivity Analysis

#### 4.8.1. Parameter Estimates

Due to the limited data for important parameters related to the natural history of *S. suis* (*β*, *σ,* and *p*), we conducted a univariable sensitivity analysis to examine how variation in these parameters influenced our model outcomes. The peer-reviewed literature did not provide meaningful ranges for these parameters. Therefore, we tested equal increases and decreases for the parameter estimates. For our transmission coefficient (*β*), we examined a range of 0.25–1.91, 3.6–6.00 weeks for the latent period (*σ)* and 0.01–0.43 for the probability of developing clinical signs (*p*). From our analyses, we obtained the range of the maximum cumulative cases and recorded the change in cases compared to our base model scenario.

#### 4.8.2. Initial Conditions 

The model assumes that the start of the infection process is initiated with the introduction of a single sub-clinically infected pig (*E0* = 1). While most pigs can become colonized with *S. suis* during or shortly after birth via the sow, the impact of vertical transmission on herd health remains unclear, since it is not known whether the same pathotypes involved in clinical *S. suis* cases had also been the early colonizers of the vaginal tract [50]. For this reason, the model does not assume that all pigs are sub-clinically infected with a pathogenic strain of *S. suis* at the start of the infection process. However, several studies have reported that the number of *S. suis* sub-clinical pigs are known to vary across farms [1], therefore we conducted an additional sensitivity analysis by varying the initial conditions for *E0* while balancing the number of pigs entering into the system in both WF and BMS.

## 5. Conclusions

Models that evaluate management practices while incorporating the biological and epidemiological aspects of this disease are critically lacking in the *S. suis* literature. While our model showed that batch-level separation may have the potential to be effective in reducing *S. suis* prevalence in the nursery, our study depends on assumptions that could be strengthened by additional research in this area, specifically using both experimental and field studies. Given that modifications in swine management practices can be complex, the objective of our study was to develop a model that could be used to demonstrate that further investigation into the adoption of such systems and practices may be warranted during a time where effective long-term strategies for disease control are limited. 

## Figures and Tables

**Table 1:** Model simulations of evaluated intervention scenarios with mean monthly incidence, mean cumulative cases counts, and percent change in cumulative cases (%) compared to the base model scenario.

Scenario	System ^1^	Disease-removal Rate, *d_2_* (days)^2^	Mean Monthly Incidence, Range	Mean Cumulative Incidence ± SD^3^	∆ cumulative cases (%)
Base model	WF	2.00	49 (7–78)	345 ± 67	–
1	BMS	2.00	24 (21–26)	141 ± 47	−59
2	WF	0.25	18 (1–37)	125 ± 57	-64
3	WF	0.50	30 (2–57)	212 ± 73	−39
4	WF	1.00	42 (4–71)	295 ± 70	−14
5	BMS	0.50	8 (7–11)	54 ± 31	−84

^1^ WF: Weekly farrowing system, BMS: Batch management system; ^2^ *d_2_*: disease-removal rate of clinically infected pigs; ^3^ SD: standard deviation. The base model scenario is shaded in grey and serves as the reference scenario for comparison to the intervention scenarios (1–5).

**Table 2:** *SEI* model transitions, events, and rates.

Transition	Event	Transition Rate
(S, E, I) → (S+1, E, I)	Recruitment of susceptible pigs	*b*
(S, E, I) → (S-1, E+1, I)	Infection of a susceptible pig	*β* *S*I
(S, E, I) → (S, E-1, I+1)	Sub-clinically infected pig develops clinical signs	(*p*)σ*E
(S, E, I) → (S, E+1, I)	Sub-clinically infected pig remains sub-clinical	(1 – *p*)σ*E
(S, E, I) → (S-1, E, I)	Production removal of susceptible pig	d_1_*S
(S, E, I) → (S, E-1, I)	Production removal of sub-clinically infected pig	d_1_*E
(S, E, I) → (S, E, I-1)	Disease-removal of clinically infected pigs	d_2_*I

**Table 3:** Parameter definitions and values used to describe the *SEI* model events.

**Parameter**	**Definition**	**Value**	**References**
*β*	Transmission coefficient for *I*	1.08	Calculated
*R_0_*	Basic reproductive number	1.4	[47]
*D*	Duration of infectiousness	1.8 weeks	Fitted
*σ*	Duration of latent period	4.8 weeks	Fitted
*p*	Probability of becoming clinically infected	0.22	Fitted
*1 – p*	Probability of remaining sub-clinically infected	0.78	Fitted
*b*	Recruitment rate of susceptible pigs	100 pigs/week	[45]
*d_1_*	Production removal rates for *S* and *E*	6 weeks	[45]
*d_2_*	Disease-removal rates for *I*	2.00 days	Assumed

**Table 4:** Evaluated intervention scenarios with corresponding batch size, disease removal rate (*d_2_*), and frequency of entry into the nursery.

Scenario	Farrowing System ^1^	Batch Size (no. of pigs)	Disease-Removal Rate, *d_2_* (days)^2^	Entry into Nursery
Base model	WF	100	2.00	Weekly
1	BMS	400	2.00	Monthly
2	WF	100	0.25	Weekly
3	WF	100	0.50	Weekly
4	WF	100	1.00	Weekly
5	BMS	400	0.50	Monthly

^1^: WF: Weekly farrowing and BMS: Batch management system; ^2^: *d_2_*: disease removal rate of clinically infected pigs. The base model scenario is shaded in grey and serves as the reference scenario for comparison to the intervention scenarios (1–5).
